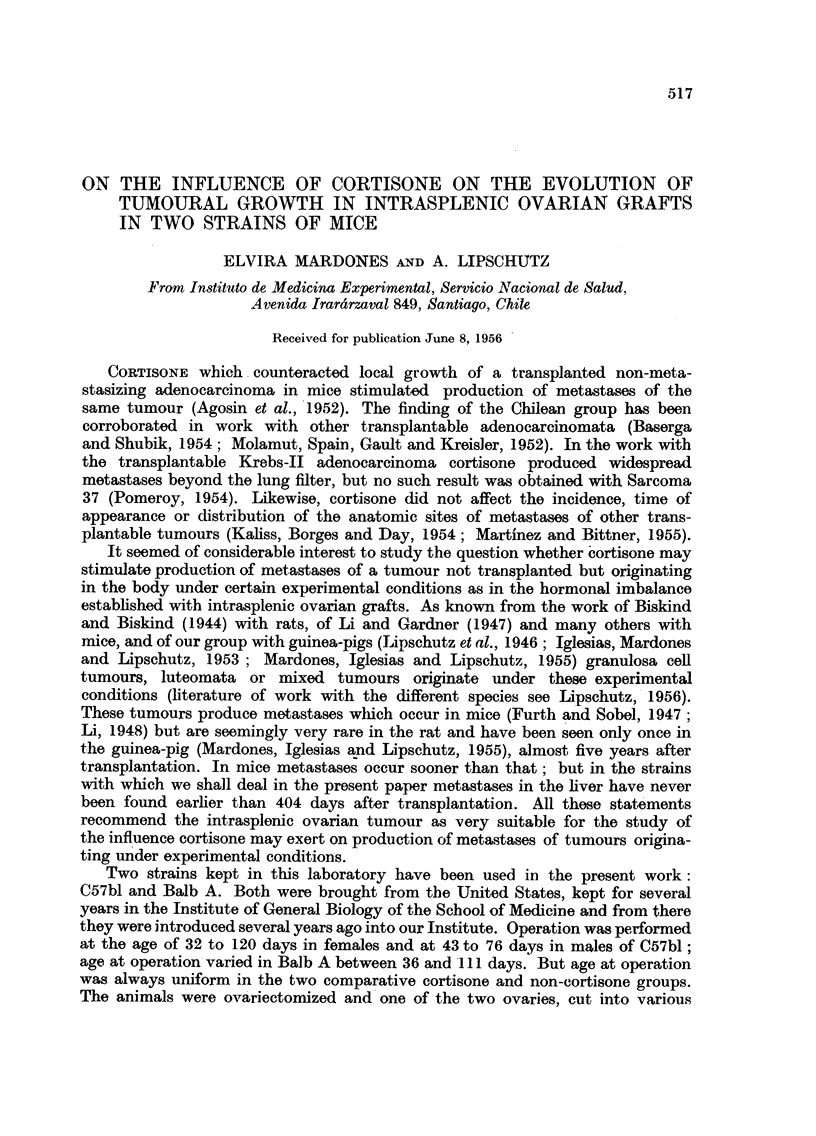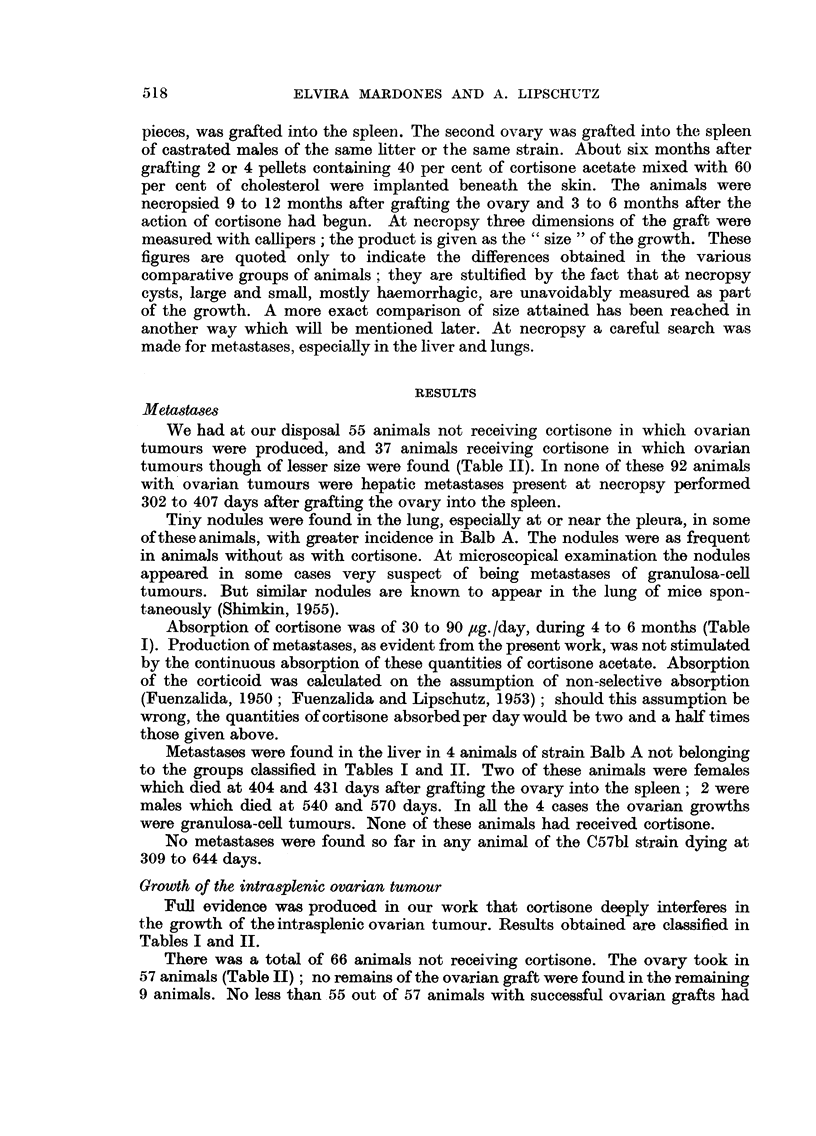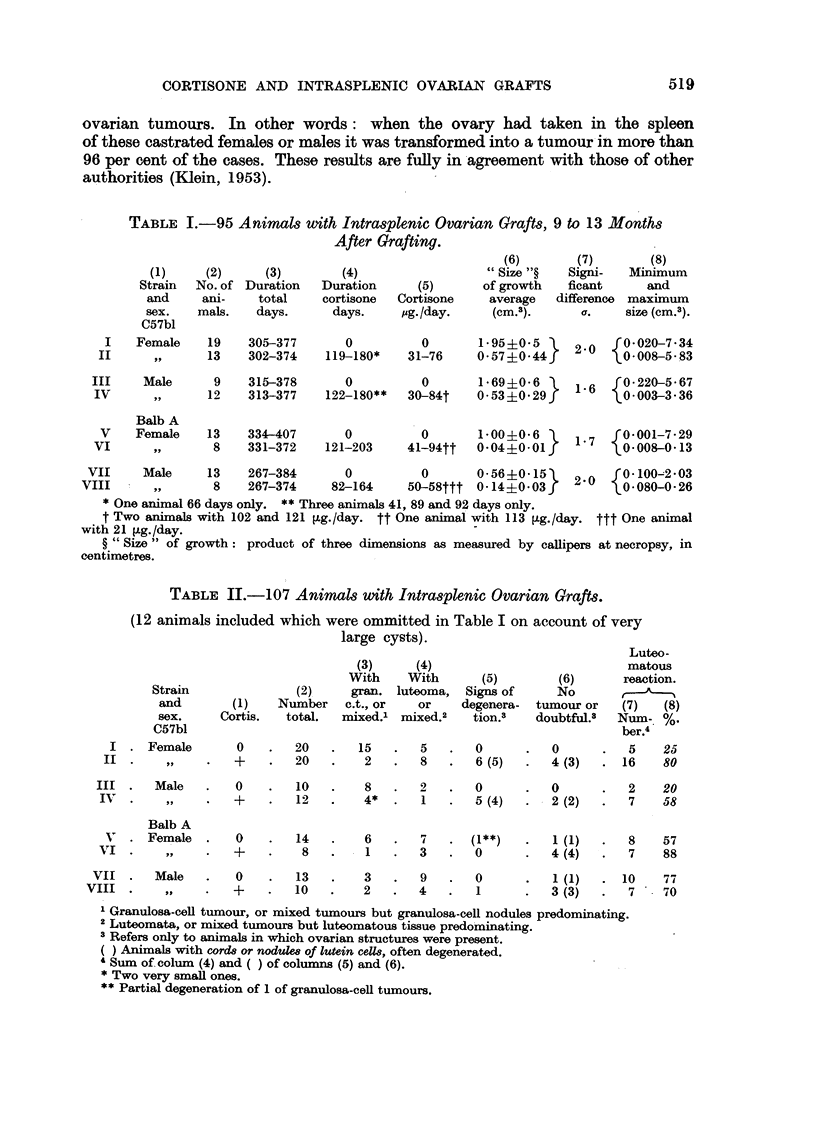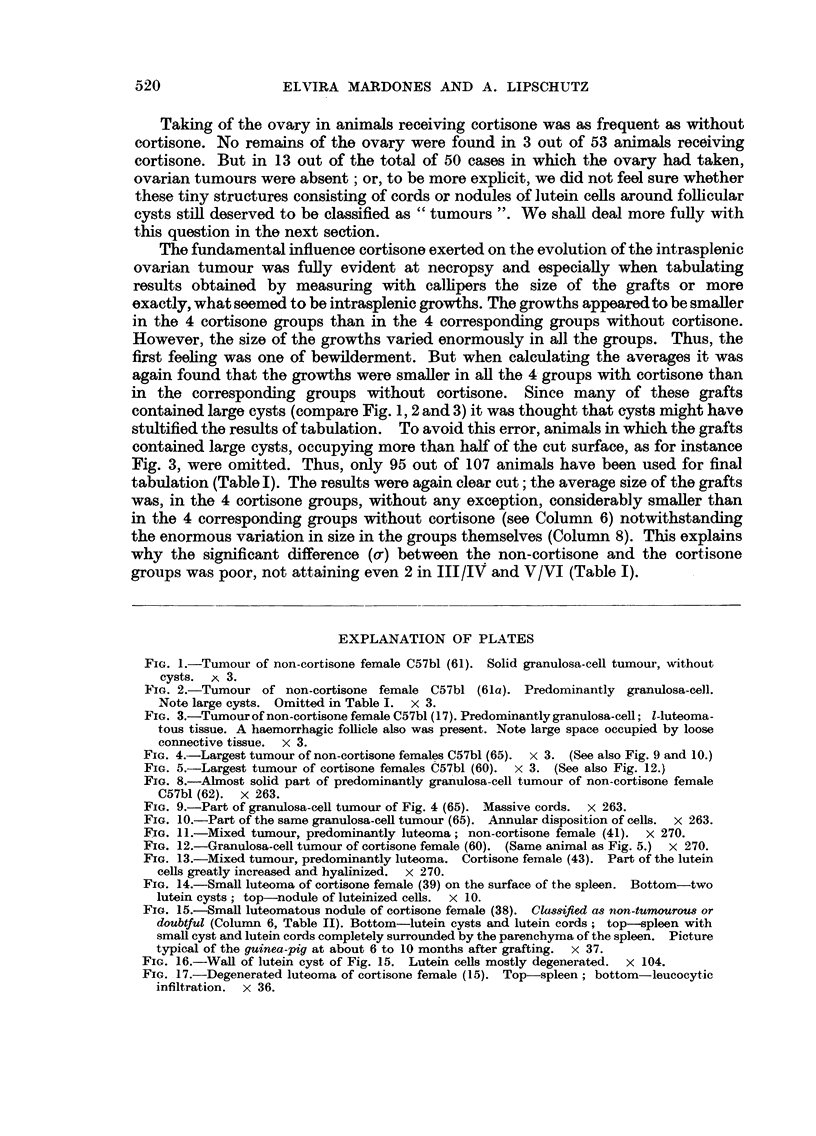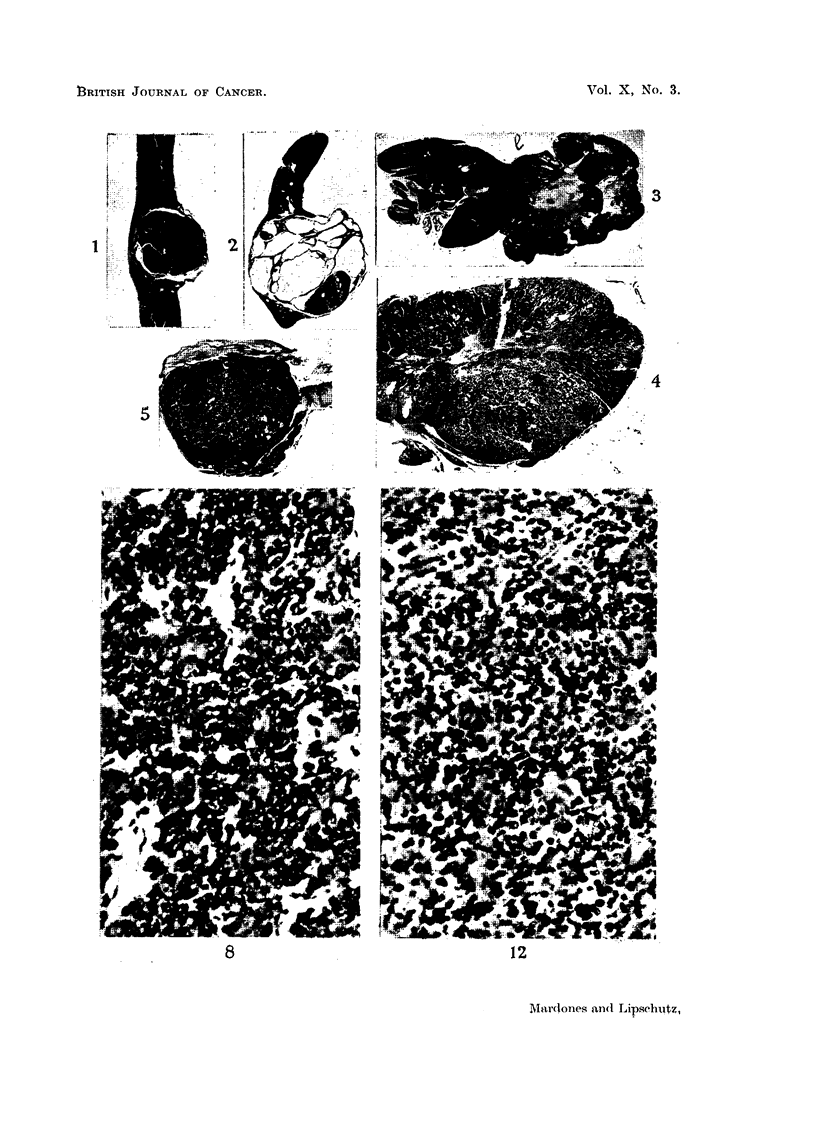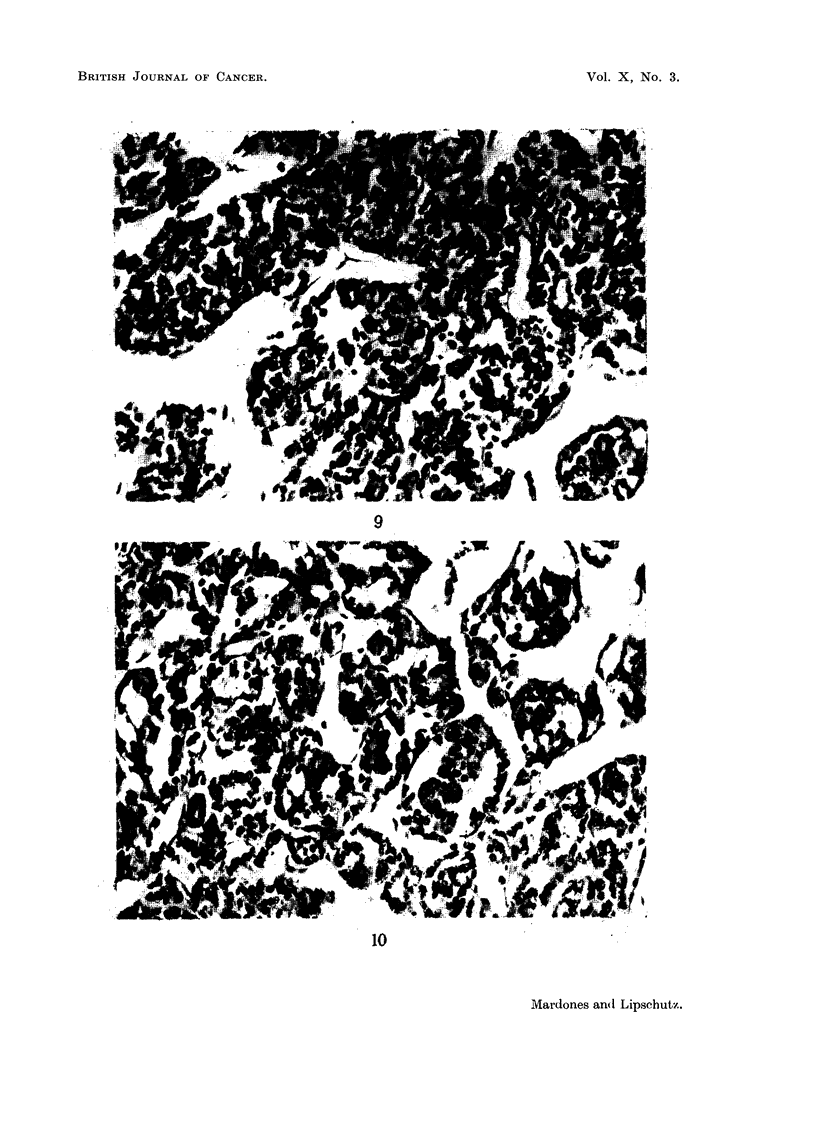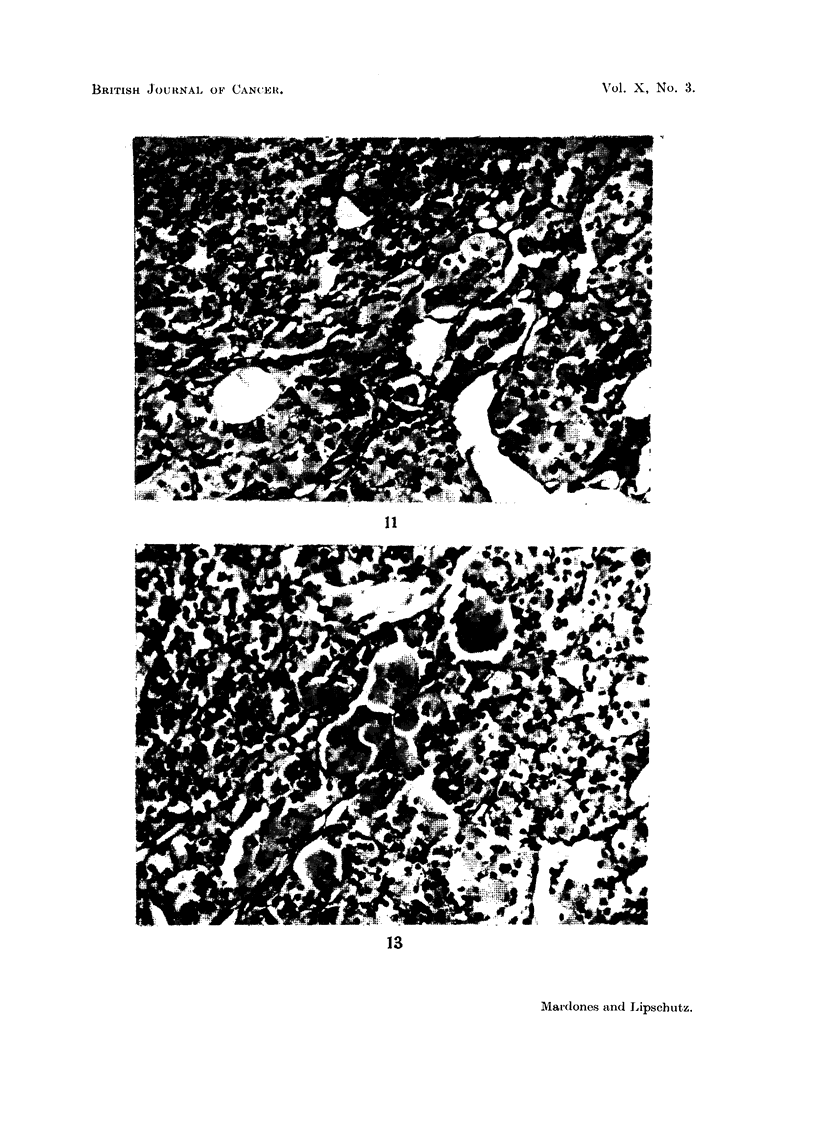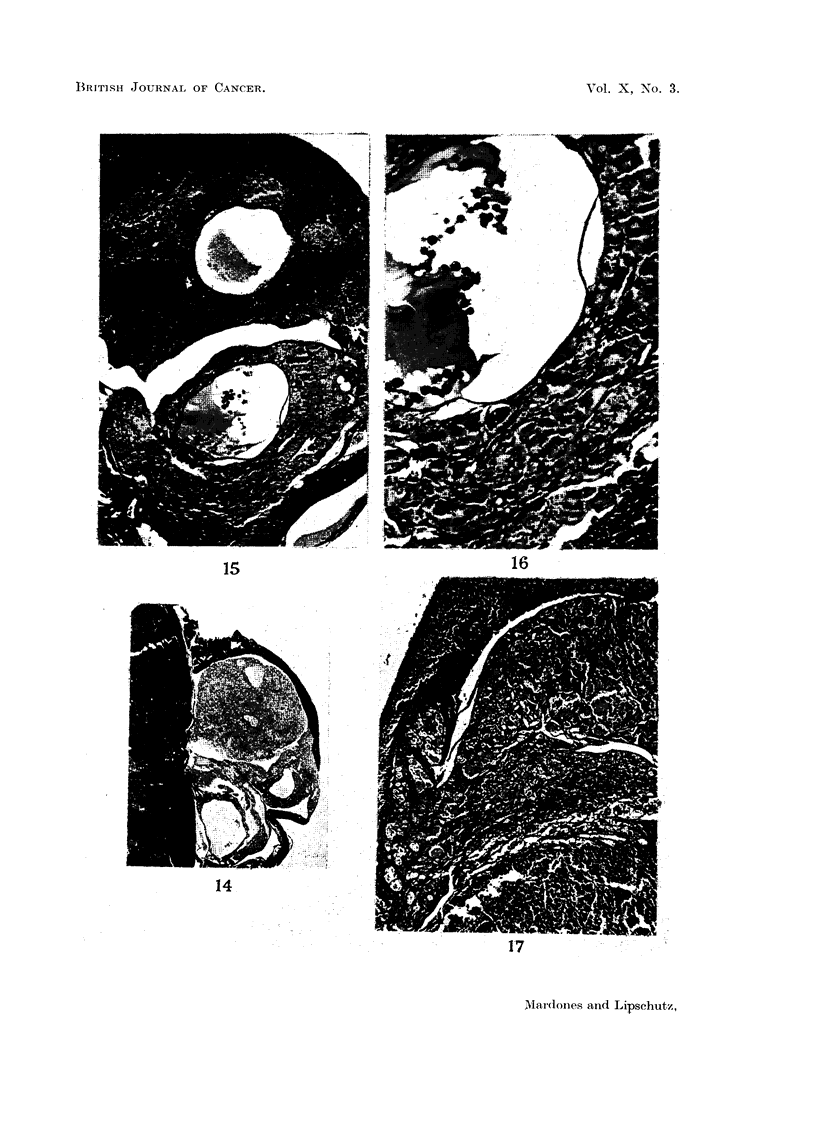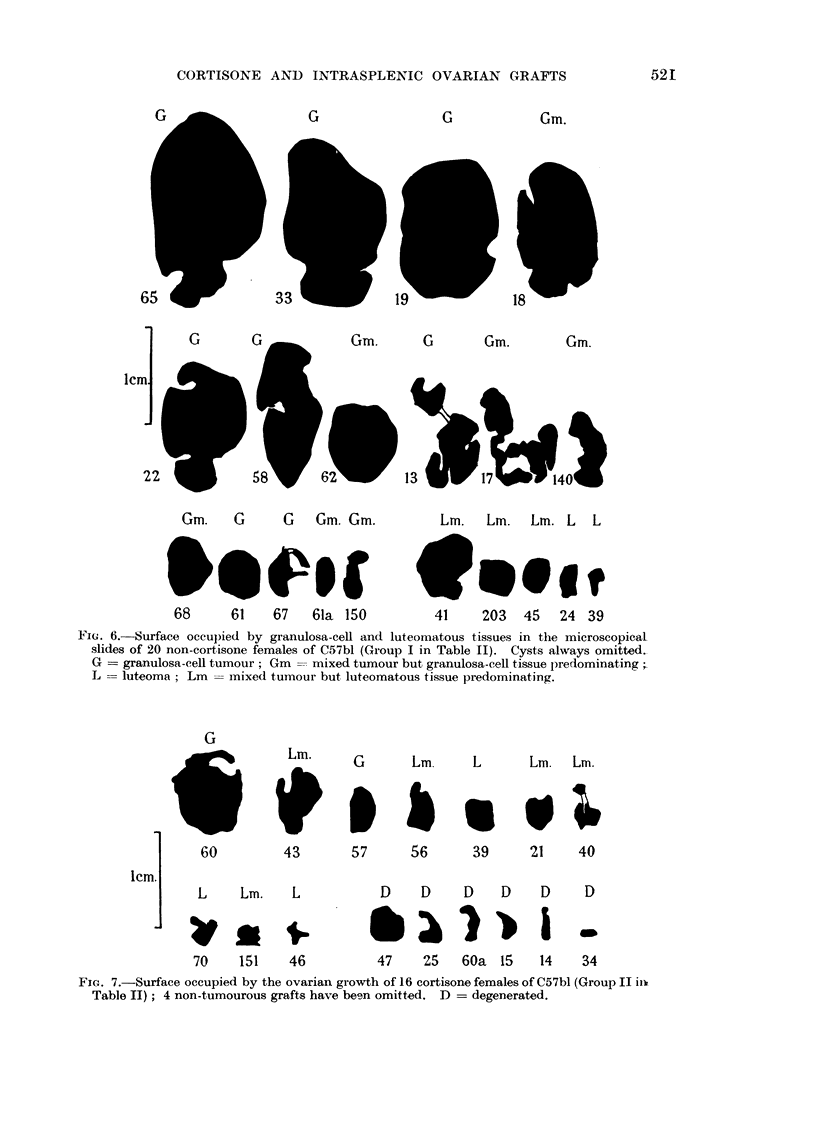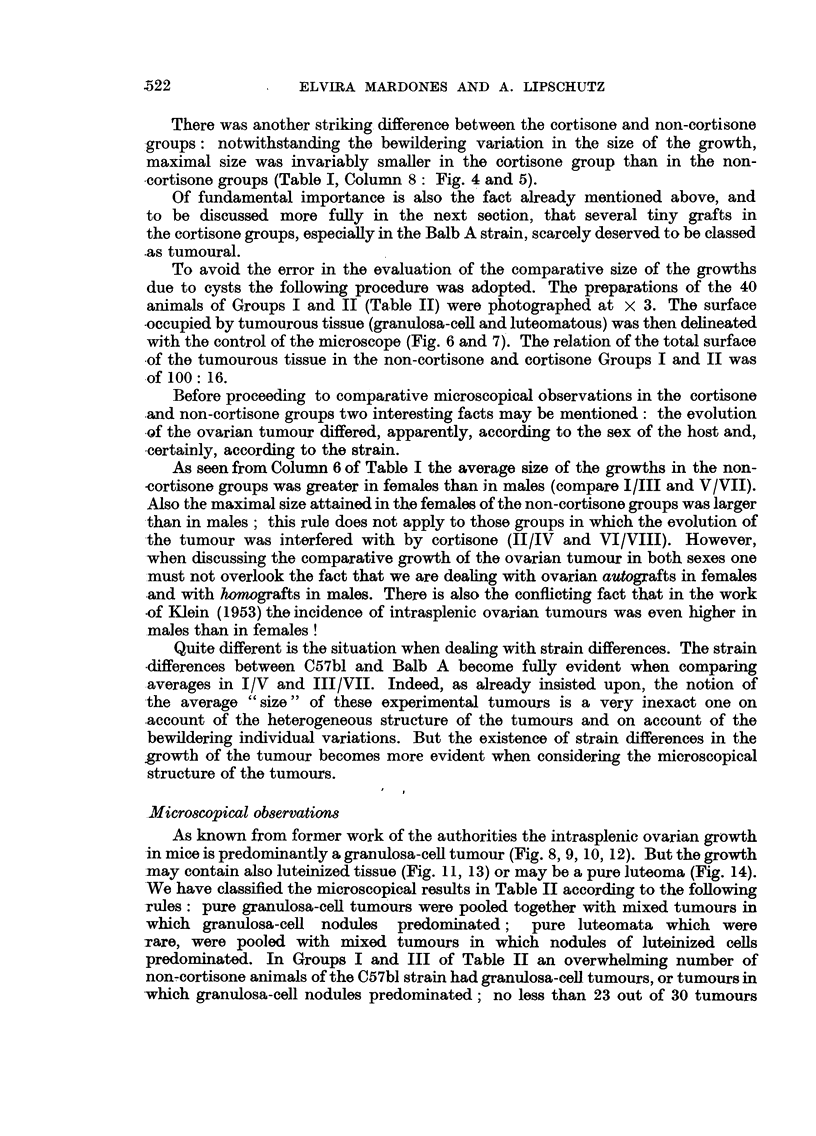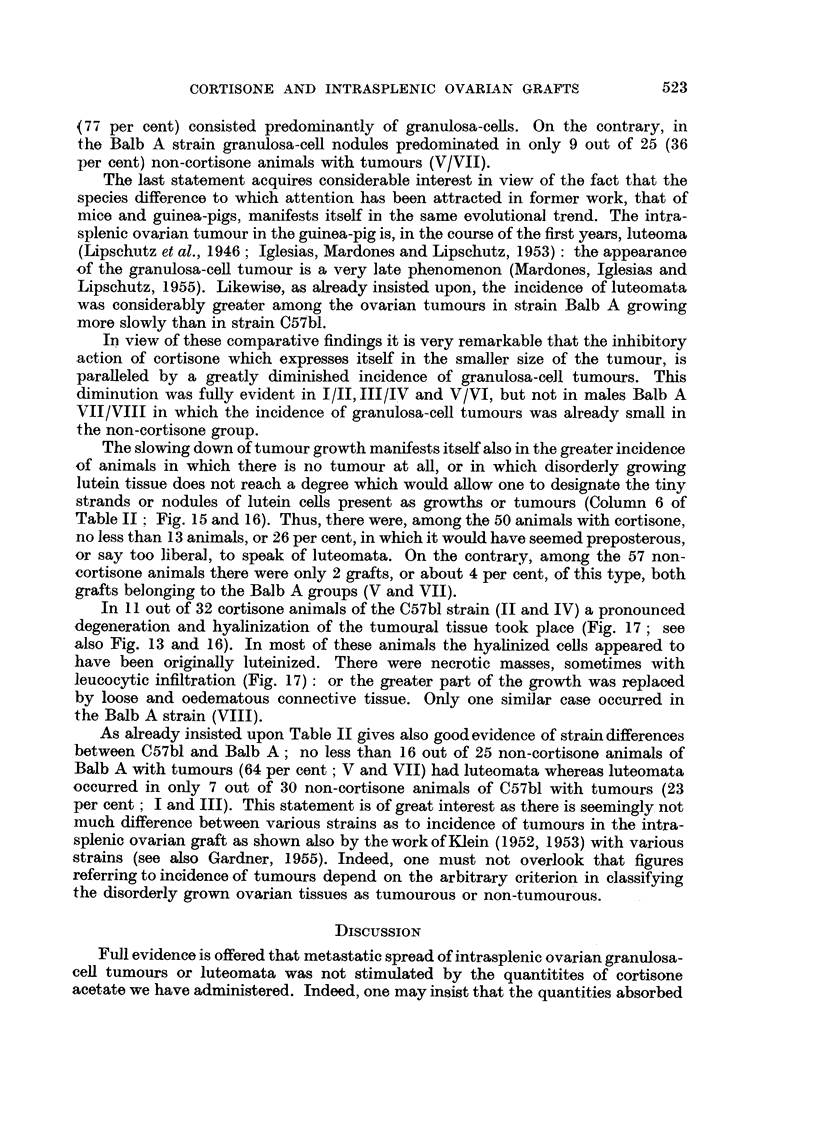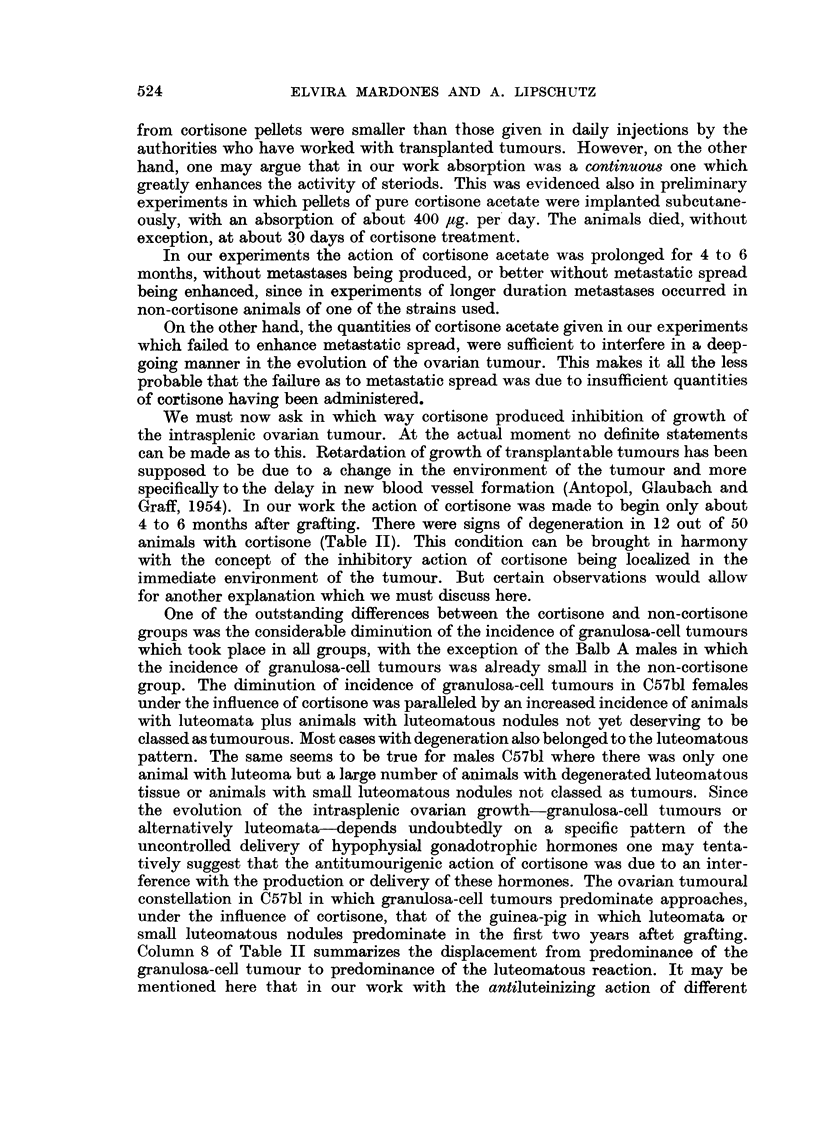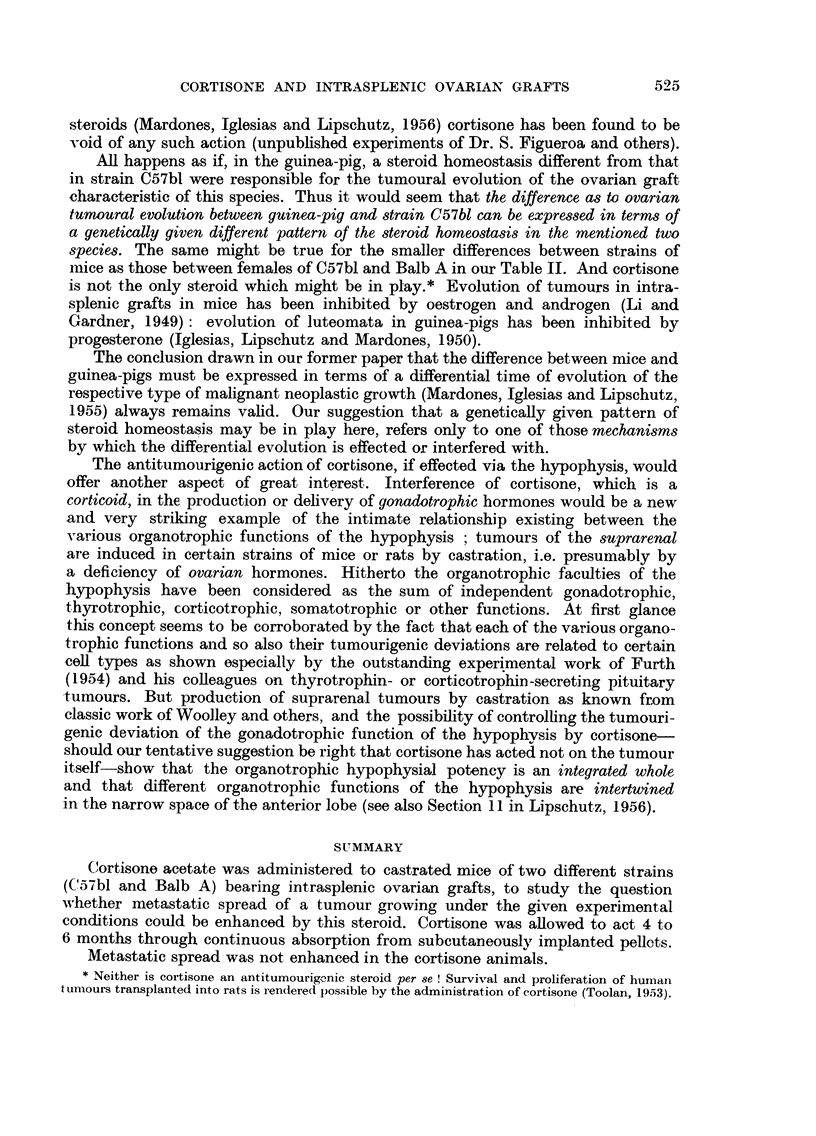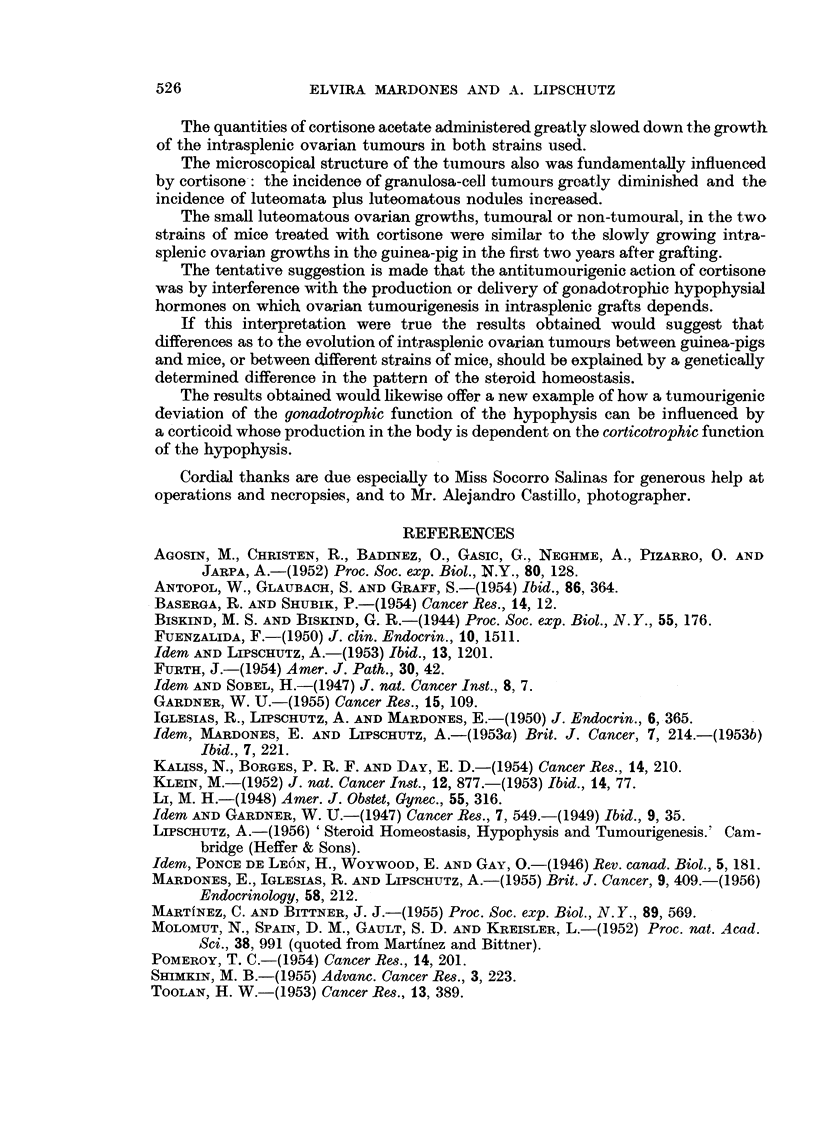# On the Influence of Cortisone on the Evolution of Tumoural Growth in Intrasplenic Ovarian Grafts in Two Strains of Mice

**DOI:** 10.1038/bjc.1956.60

**Published:** 1956-09

**Authors:** Elvira Mardones, A. Lipschutz

## Abstract

**Images:**


### ON THE INFLUENCE OF CORTISONE ON THE EVOLUTION OF TUMOURAL GROWTH IN INTRASPLENIC OVARIAN GRAFTS IN TWO STRAINS OF MICE

ELVIRA MARDONES AND A. LIPSCHUTZ

*From Instituto de Medicina Experimental, Servicio Nacional de Salud, Avenida Irarárzaval* 849, *Santiago, Chile*

Received for publication June 8, 1956

CORTISONE which counteracted local growth of a transplanted non-metastasizing adenocarcinoma in mice stimulated production of metastases of the same tumour (Agosin *et al.*, 1952). The finding of the Chilean group has been corroborated in work with other transplantable adenocarcinomata (Baserga and Shubik, 1954 ; Molamut, Spain, Gault and Kreisler, 1952). In the work with the transplantable Krebs-II adenocarcinoma cortisone produced widespread metastases beyond the lung filter, but no such result was obtained with Sarcoma 37 (Pomeroy, 1954). Likewise, cortisone did not affect the incidence, time of appearance or distribution of the anatomic sites of metastases of other transplantable tumours (Kaliss, Borges and Day, 1954 ; Martínez and Bittner, 1955).

It seemed of considerable interest to study the question whether cortisone may stimulate production of metastases of a tumour not transplanted but originating in the body under certain experimental conditions as in the hormonal imbalance established with intrasplenic ovarian grafts. As known from the work of Biskind and Biskind (1944) with rats, of Li and Gardner (1947) and many others with mice, and of our group with guinea-pigs (Lipschutz *et al.*, 1946 ; Iglesias, Mardones and Lipschutz, 1953 ; Mardones, Iglesias and Lipschutz, 1955) granulosa cell tumours, luteomata or mixed tumours originate under these experimental conditions (literature of work with the different species see Lipschutz, 1956). These tumours produce metastases which occur in mice (Furth and Sobel, 1947 ; Li, 1948) but are seemingly very rare in the rat and have been seen only once in the guinea-pig (Mardones, Iglesias and Lipschutz, 1955), almost five years after transplantation. In mice metastases occur sooner than that ; but in the strains with which we shall deal in the present paper metastases in the liver have never been found earlier than 404 days after transplantation. All these statements recommend the intrasplenic ovarian tumour as very suitable for the study of the influence cortisone may exert on production of metastases of tumours originating under experimental conditions.

Two strains kept in this laboratory have been used in the present work : C57bl and Balb A. Both were brought from the United States, kept for several years in the Institute of General Biology of the School of Medicine and from there they were introduced several years ago into our Institute. Operation was performed at the age of 32 to 120 days in females and at 43 to 76 days in males of C57bl ; age at operation varied in Balb A between 36 and 111 days. But age at operation was always uniform in the two comparative cortisone and non-cortisone groups. The animals were ovariectomized and one of the two ovaries, cut into various pieces, was grafted into the spleen. The second ovary was grafted into the spleen of castrated males of the same litter or the same strain. About six months after grafting 2 or 4 pellets containing 40 per cent of cortisone acetate mixed with 60 per cent of cholesterol were implanted beneath the skin. The animals were necropsied 9 to 12 months after grafting the ovary and 3 to 6 months after the action of cortisone had begun. At necropsy three dimensions of the graft were measured with callipers ; the product is given as the " size " of the growth. These figures are quoted only to indicate the differences obtained in the various comparative groups of animals ; they are stultified by the fact that at necropsy cysts, large and small, mostly haemorrhagic, are unavoidably measured as part of the growth. A more exact comparison of size attained has been reached in another way which will be mentioned later. At necropsy a careful search was made for metastases, especially in the liver and lungs.

### RESULTS

*Metastases*

We had at our disposal 55 animals not receiving cortisone in which ovarian tumours were produced, and 37 animals receiving cortisone in which ovarian tumours though of lesser size were found (Table II). In none of these 92 animals with ovarian tumours were hepatic metastases present at necropsy performed 302 to 407 days after grafting the ovary into the spleen.

Tiny nodules were found in the lung, especially at or near the pleura, in some of these animals, with greater incidence in Balb A. The nodules were as frequent in animals without as with cortisone. At microscopical examination the nodules appeared in some cases very suspect of being metastases of granulosa-cell tumours. But similar nodules are known to appear in the lung of mice spontaneously (Shimkin, 1955).

Absorption of cortisone was of 30 to 90 $\mu$g./day, during 4 to 6 months (Table I). Production of metastases, as evident from the present work, was not stimulated by the continuous absorption of these quantities of cortisone acetate. Absorption of the corticoid was calculated on the assumption of non-selective absorption (Fuenzalida, 1950 ; Fuenzalida and Lipschutz, 1953) ; should this assumption be wrong, the quantities of cortisone absorbed per day would be two and a half times those given above.

Metastases were found in the liver in 4 animals of strain Balb A not belonging to the groups classified in Tables I and II. Two of these animals were females which died at 404 and 431 days after grafting the ovary into the spleen ; 2 were males which died at 540 and 570 days. In all the 4 cases the ovarian growths were granulosa-cell tumours. None of these animals had received cortisone.

No metastases were found so far in any animal of the C57bl strain dying at 309 to 644 days.

*Growth of the intrasplenic ovarian tumour*

Full evidence was produced in our work that cortisone deeply interferes in the growth of the intrasplenic ovarian tumour. Results obtained are classified in Tables I and II.

There was a total of 66 animals not receiving cortisone. The ovary took in 57 animals (Table II) ; no remains of the ovarian graft were found in the remaining 9 animals. No less than 55 out of 57 animals with successful ovarian grafts had ovarian tumours. In other words : when the ovary had taken in the spleen of these castrated females or males it was transformed into a tumour in more than 96 per cent of the cases. These results are fully in agreement with those of other authorities (Klein, 1953).

TABLE I.—95 *Animals with Intrasplenic Ovarian Grafts,* 9 *to* 13 *Months After Grafting.*

| | (1) Strain and sex. C57bl | (2) No. of animals. | (3) Duration total days. | (4) Duration cortisone days. | (5) Cortisone µg./day. | (6) " Size "§ of growth average (cm.³). | (7) Significant difference σ. | (8) Minimum and maximum size (cm.³). |
|---|---|---|---|---|---|---|---|---|
| I | Female | 19 | 305–377 | 0 | 0 | 1·95±0·5 | 2·0 | 0·020–7·34 |
| II | ,, | 13 | 302–374 | 119–180* | 31–76 | 0·57±0·44 | | 0·008–5·83 |
| III | Male | 9 | 315–378 | 0 | 0 | 1·69±0·6 | 1·6 | 0·220–5·67 |
| IV | ,, | 12 | 313–377 | 122–180** | 30–84† | 0·53±0·29 | | 0·003–3·36 |
| | Balb A | | | | | | | |
| V | Female | 13 | 334–407 | 0 | 0 | 1·00±0·6 | 1·7 | 0·001–7·29 |
| VI | ,, | 8 | 331–372 | 121–203 | 41–94†† | 0·04±0·01 | | 0·008–0·13 |
| VII | Male | 13 | 267–384 | 0 | 0 | 0·56±0·15 | 2·0 | 0·100–2·03 |
| VIII | ,, | 8 | 267–374 | 82–164 | 50–58††† | 0·14±0·03 | | 0·080–0·26 |

* One animal 66 days only. ** Three animals 41, 89 and 92 days only.

† Two animals with 102 and 121 µg./day. †† One animal with 113 µg./day. ††† One animal with 21 µg./day.

§ " Size " of growth : product of three dimensions as measured by callipers at necropsy, in centimetres.

TABLE II.—107 *Animals with Intrasplenic Ovarian Grafts.*

(12 animals included which were ommitted in Table I on account of very large cysts).

| | Strain and sex. C57bl | (1) Cortis. | (2) Number total. | (3) With gran. c.t., or mixed.¹ | (4) With luteoma, or mixed.² | (5) Signs of degeneration.³ | (6) No tumour or doubtful.³ | Luteomatous reaction. (7) Number.⁴ | (8) %. |
|---|---|---|---|---|---|---|---|---|---|
| I | Female | 0 | 20 | 15 | 5 | 0 | 0 | 5 | *25* |
| II | ,, | + | 20 | 2 | 8 | 6 (5) | 4 (3) | 16 | *80* |
| III | Male | 0 | 10 | 8 | 2 | 0 | 0 | 2 | *20* |
| IV | ,, | + | 12 | 4* | 1 | 5 (4) | 2 (2) | 7 | *58* |
| | Balb A | | | | | | | | |
| V | Female | 0 | 14 | 6 | 7 | (1**) | 1 (1) | 8 | 57 |
| VI | ,, | + | 8 | 1 | 3 | 0 | 4 (4) | 7 | 88 |
| VII | Male | 0 | 13 | 3 | 9 | 0 | 1 (1) | 10 | 77 |
| VIII | ,, | + | 10 | 2 | 4 | 1 | 3 (3) | 7 | 70 |

¹ Granulosa-cell tumour, or mixed tumours but granulosa-cell nodules predominating.
² Luteomata, or mixed tumours but luteomatous tissue predominating.
³ Refers only to animals in which ovarian structures were present.
( ) Animals with *cords or nodules of lutein cells,* often degenerated.
⁴ Sum of colum (4) and ( ) of columns (5) and (6).
* Two very small ones.
** Partial degeneration of 1 of granulosa-cell tumours.

Taking of the ovary in animals receiving cortisone was as frequent as without cortisone. No remains of the ovary were found in 3 out of 53 animals receiving cortisone. But in 13 out of the total of 50 cases in which the ovary had taken, ovarian tumours were absent ; or, to be more explicit, we did not feel sure whether these tiny structures consisting of cords or nodules of lutein cells around follicular cysts still deserved to be classified as " tumours ". We shall deal more fully with this question in the next section.

The fundamental influence cortisone exerted on the evolution of the intrasplenic ovarian tumour was fully evident at necropsy and especially when tabulating results obtained by measuring with callipers the size of the grafts or more exactly, what seemed to be intrasplenic growths. The growths appeared to be smaller in the 4 cortisone groups than in the 4 corresponding groups without cortisone. However, the size of the growths varied enormously in all the groups. Thus, the first feeling was one of bewilderment. But when calculating the averages it was again found that the growths were smaller in all the 4 groups with cortisone than in the corresponding groups without cortisone. Since many of these grafts contained large cysts (compare Fig. 1, 2 and 3) it was thought that cysts might have stultified the results of tabulation. To avoid this error, animals in which the grafts contained large cysts, occupying more than half of the cut surface, as for instance Fig. 3, were omitted. Thus, only 95 out of 107 animals have been used for final tabulation (Table I). The results were again clear cut ; the average size of the grafts was, in the 4 cortisone groups, without any exception, considerably smaller than in the 4 corresponding groups without cortisone (see Column 6) notwithstanding the enormous variation in size in the groups themselves (Column 8). This explains why the significant difference ($\sigma$) between the non-cortisone and the cortisone groups was poor, not attaining even 2 in III/IV and V/VI (Table I).

---

### EXPLANATION OF PLATES

FIG. 1.—Tumour of non-cortisone female C57bl (61). Solid granulosa-cell tumour, without cysts. × 3.

FIG. 2.—Tumour of non-cortisone female C57bl (61*a*). Predominantly granulosa-cell. Note large cysts. Omitted in Table I. × 3.

FIG. 3.—Tumour of non-cortisone female C57bl (17). Predominantly granulosa-cell; *l*-luteomatous tissue. A haemorrhagic follicle also was present. Note large space occupied by loose connective tissue. × 3.

FIG. 4.—Largest tumour of non-cortisone females C57bl (65). × 3. (See also Fig. 9 and 10.)

FIG. 5.—Largest tumour of cortisone females C57bl (60). × 3. (See also Fig. 12.)

FIG. 8.—Almost solid part of predominantly granulosa-cell tumour of non-cortisone female C57bl (62). × 263.

FIG. 9.—Part of granulosa-cell tumour of Fig. 4 (65). Massive cords. × 263.

FIG. 10.—Part of the same granulosa-cell tumour (65). Annular disposition of cells. × 263.

FIG. 11.—Mixed tumour, predominantly luteoma ; non-cortisone female (41). × 270.

FIG. 12.—Granulosa-cell tumour of cortisone female (60). (Same animal as Fig. 5.) × 270.

FIG. 13.—Mixed tumour, predominantly luteoma. Cortisone female (43). Part of the lutein cells greatly increased and hyalinized. × 270.

FIG. 14.—Small luteoma of cortisone female (39) on the surface of the spleen. Bottom—two lutein cysts ; top—nodule of luteinized cells. × 10.

FIG. 15.—Small luteomatous nodule of cortisone female (38). *Classified as non-tumourous or doubtful* (Column 6, Table II). Bottom—lutein cysts and lutein cords ; top—spleen with small cyst and lutein cords completely surrounded by the parenchyma of the spleen. Picture typical of the *guinea-pig* at about 6 to 10 months after grafting. × 37.

FIG. 16.—Wall of lutein cyst of Fig. 15. Lutein cells mostly degenerated. × 104.

FIG. 17.—Degenerated luteoma of cortisone female (15). Top—spleen ; bottom—leucocytic infiltration. × 36.

Mardones and Lipschutz.

9

10

Mardones and Lipschutz.

11

13

Mardones and Lipschutz.

15

16

14

17

Mardones and Lipschutz.

FIG. 6.—Surface occupied by granulosa-cell and luteomatous tissues in the microscopical slides of 20 non-cortisone females of C57bl (Group I in Table II). Cysts always omitted. G = granulosa-cell tumour ; Gm = mixed tumour but granulosa-cell tissue predominating ; L = luteoma ; Lm = mixed tumour but luteomatous tissue predominating.

FIG. 7.—Surface occupied by the ovarian growth of 16 cortisone females of C57bl (Group II in Table II) ; 4 non-tumourous grafts have been omitted. D = degenerated.

There was another striking difference between the cortisone and non-cortisone groups: notwithstanding the bewildering variation in the size of the growth, maximal size was invariably smaller in the cortisone group than in the non-cortisone groups (Table I, Column 8 : Fig. 4 and 5).

Of fundamental importance is also the fact already mentioned above, and to be discussed more fully in the next section, that several tiny grafts in the cortisone groups, especially in the Balb A strain, scarcely deserved to be classed as tumoural.

To avoid the error in the evaluation of the comparative size of the growths due to cysts the following procedure was adopted. The preparations of the 40 animals of Groups I and II (Table II) were photographed at $\times$ 3. The surface occupied by tumourous tissue (granulosa-cell and luteomatous) was then delineated with the control of the microscope (Fig. 6 and 7). The relation of the total surface of the tumourous tissue in the non-cortisone and cortisone Groups I and II was of 100 : 16.

Before proceeding to comparative microscopical observations in the cortisone and non-cortisone groups two interesting facts may be mentioned: the evolution of the ovarian tumour differed, apparently, according to the sex of the host and, certainly, according to the strain.

As seen from Column 6 of Table I the average size of the growths in the non-cortisone groups was greater in females than in males (compare I/III and V/VII). Also the maximal size attained in the females of the non-cortisone groups was larger than in males ; this rule does not apply to those groups in which the evolution of the tumour was interfered with by cortisone (II/IV and VI/VIII). However, when discussing the comparative growth of the ovarian tumour in both sexes one must not overlook the fact that we are dealing with ovarian *auto*grafts in females and with *homo*grafts in males. There is also the conflicting fact that in the work of Klein (1953) the incidence of intrasplenic ovarian tumours was even higher in males than in females !

Quite different is the situation when dealing with strain differences. The strain differences between C57bl and Balb A become fully evident when comparing averages in I/V and III/VII. Indeed, as already insisted upon, the notion of the average "size" of these experimental tumours is a very inexact one on account of the heterogeneous structure of the tumours and on account of the bewildering individual variations. But the existence of strain differences in the growth of the tumour becomes more evident when considering the microscopical structure of the tumours.

*Microscopical observations*

As known from former work of the authorities the intrasplenic ovarian growth in mice is predominantly a granulosa-cell tumour (Fig. 8, 9, 10, 12). But the growth may contain also luteinized tissue (Fig. 11, 13) or may be a pure luteoma (Fig. 14). We have classified the microscopical results in Table II according to the following rules: pure granulosa-cell tumours were pooled together with mixed tumours in which granulosa-cell nodules predominated; pure luteomata which were rare, were pooled with mixed tumours in which nodules of luteinized cells predominated. In Groups I and III of Table II an overwhelming number of non-cortisone animals of the C57bl strain had granulosa-cell tumours, or tumours in which granulosa-cell nodules predominated ; no less than 23 out of 30 tumours

(77 per cent) consisted predominantly of granulosa-cells. On the contrary, in the Balb A strain granulosa-cell nodules predominated in only 9 out of 25 (36 per cent) non-cortisone animals with tumours (V/VII).

The last statement acquires considerable interest in view of the fact that the species difference to which attention has been attracted in former work, that of mice and guinea-pigs, manifests itself in the same evolutional trend. The intrasplenic ovarian tumour in the guinea-pig is, in the course of the first years, luteoma (Lipschutz *et al.*, 1946 ; Iglesias, Mardones and Lipschutz, 1953) : the appearance of the granulosa-cell tumour is a very late phenomenon (Mardones, Iglesias and Lipschutz, 1955). Likewise, as already insisted upon, the incidence of luteomata was considerably greater among the ovarian tumours in strain Balb A growing more slowly than in strain C57bl.

In view of these comparative findings it is very remarkable that the inhibitory action of cortisone which expresses itself in the smaller size of the tumour, is paralleled by a greatly diminished incidence of granulosa-cell tumours. This diminution was fully evident in I/II, III/IV and V/VI, but not in males Balb A VII/VIII in which the incidence of granulosa-cell tumours was already small in the non-cortisone group.

The slowing down of tumour growth manifests itself also in the greater incidence of animals in which there is no tumour at all, or in which disorderly growing lutein tissue does not reach a degree which would allow one to designate the tiny strands or nodules of lutein cells present as growths or tumours (Column 6 of Table II ; Fig. 15 and 16). Thus, there were, among the 50 animals with cortisone, no less than 13 animals, or 26 per cent, in which it would have seemed preposterous, or say too liberal, to speak of luteomata. On the contrary, among the 57 non-cortisone animals there were only 2 grafts, or about 4 per cent, of this type, both grafts belonging to the Balb A groups (V and VII).

In 11 out of 32 cortisone animals of the C57bl strain (II and IV) a pronounced degeneration and hyalinization of the tumoural tissue took place (Fig. 17 ; see also Fig. 13 and 16). In most of these animals the hyalinized cells appeared to have been originally luteinized. There were necrotic masses, sometimes with leucocytic infiltration (Fig. 17) : or the greater part of the growth was replaced by loose and oedematous connective tissue. Only one similar case occurred in the Balb A strain (VIII).

As already insisted upon Table II gives also good evidence of strain differences between C57bl and Balb A ; no less than 16 out of 25 non-cortisone animals of Balb A with tumours (64 per cent ; V and VII) had luteomata whereas luteomata occurred in only 7 out of 30 non-cortisone animals of C57bl with tumours (23 per cent ; I and III). This statement is of great interest as there is seemingly not much difference between various strains as to incidence of tumours in the intrasplenic ovarian graft as shown also by the work of Klein (1952, 1953) with various strains (see also Gardner, 1955). Indeed, one must not overlook that figures referring to incidence of tumours depend on the arbitrary criterion in classifying the disorderly grown ovarian tissues as tumourous or non-tumourous.

### Discussion

Full evidence is offered that metastatic spread of intrasplenic ovarian granulosa-cell tumours or luteomata was not stimulated by the quantitites of cortisone acetate we have administered. Indeed, one may insist that the quantities absorbed from cortisone pellets were smaller than those given in daily injections by the authorities who have worked with transplanted tumours. However, on the other hand, one may argue that in our work absorption was a *continuous* one which greatly enhances the activity of steriods. This was evidenced also in preliminary experiments in which pellets of pure cortisone acetate were implanted subcutaneously, with an absorption of about 400 $\mu$g. per day. The animals died, without exception, at about 30 days of cortisone treatment.

In our experiments the action of cortisone acetate was prolonged for 4 to 6 months, without metastases being produced, or better without metastatic spread being enhanced, since in experiments of longer duration metastases occurred in non-cortisone animals of one of the strains used.

On the other hand, the quantities of cortisone acetate given in our experiments which failed to enhance metastatic spread, were sufficient to interfere in a deep-going manner in the evolution of the ovarian tumour. This makes it all the less probable that the failure as to metastatic spread was due to insufficient quantities of cortisone having been administered.

We must now ask in which way cortisone produced inhibition of growth of the intrasplenic ovarian tumour. At the actual moment no definite statements can be made as to this. Retardation of growth of transplantable tumours has been supposed to be due to a change in the environment of the tumour and more specifically to the delay in new blood vessel formation (Antopol, Glaubach and Graff, 1954). In our work the action of cortisone was made to begin only about 4 to 6 months after grafting. There were signs of degeneration in 12 out of 50 animals with cortisone (Table II). This condition can be brought in harmony with the concept of the inhibitory action of cortisone being localized in the immediate environment of the tumour. But certain observations would allow for another explanation which we must discuss here.

One of the outstanding differences between the cortisone and non-cortisone groups was the considerable diminution of the incidence of granulosa-cell tumours which took place in all groups, with the exception of the Balb A males in which the incidence of granulosa-cell tumours was already small in the non-cortisone group. The diminution of incidence of granulosa-cell tumours in C57bl females under the influence of cortisone was paralleled by an increased incidence of animals with luteomata plus animals with luteomatous nodules not yet deserving to be classed as tumourous. Most cases with degeneration also belonged to the luteomatous pattern. The same seems to be true for males C57bl where there was only one animal with luteoma but a large number of animals with degenerated luteomatous tissue or animals with small luteomatous nodules not classed as tumours. Since the evolution of the intrasplenic ovarian growth—granulosa-cell tumours or alternatively luteomata—depends undoubtedly on a specific pattern of the uncontrolled delivery of hypophysial gonadotrophic hormones one may tentatively suggest that the antitumourigenic action of cortisone was due to an interference with the production or delivery of these hormones. The ovarian tumoural constellation in C57bl in which granulosa-cell tumours predominate approaches, under the influence of cortisone, that of the guinea-pig in which luteomata or small luteomatous nodules predominate in the first two years aftet grafting. Column 8 of Table II summarizes the displacement from predominance of the granulosa-cell tumour to predominance of the luteomatous reaction. It may be mentioned here that in our work with the *anti*luteinizing action of different steroids (Mardones, Iglesias and Lipschutz, 1956) cortisone has been found to be void of any such action (unpublished experiments of Dr. S. Figueroa and others).

All happens as if, in the guinea-pig, a steroid homeostasis different from that in strain C57bl were responsible for the tumoural evolution of the ovarian graft characteristic of this species. Thus it would seem that *the difference as to ovarian tumoural evolution between guinea-pig and strain C57bl can be expressed in terms of a genetically given different pattern of the steroid homeostasis in the mentioned two species.* The same might be true for the smaller differences between strains of mice as those between females of C57bl and Balb A in our Table II. And cortisone is not the only steroid which might be in play.* Evolution of tumours in intrasplenic grafts in mice has been inhibited by oestrogen and androgen (Li and Gardner, 1949): evolution of luteomata in guinea-pigs has been inhibited by progesterone (Iglesias, Lipschutz and Mardones, 1950).

The conclusion drawn in our former paper that the difference between mice and guinea-pigs must be expressed in terms of a differential time of evolution of the respective type of malignant neoplastic growth (Mardones, Iglesias and Lipschutz, 1955) always remains valid. Our suggestion that a genetically given pattern of steroid homeostasis may be in play here, refers only to one of those *mechanisms* by which the differential evolution is effected or interfered with.

The antitumourigenic action of cortisone, if effected via the hypophysis, would offer another aspect of great interest. Interference of cortisone, which is a *corticoid,* in the production or delivery of *gonadotrophic* hormones would be a new and very striking example of the intimate relationship existing between the various organotrophic functions of the hypophysis ; tumours of the *suprarenal* are induced in certain strains of mice or rats by castration, i.e. presumably by a deficiency of *ovarian* hormones. Hitherto the organotrophic faculties of the hypophysis have been considered as the sum of independent gonadotrophic, thyrotrophic, corticotrophic, somatotrophic or other functions. At first glance this concept seems to be corroborated by the fact that each of the various organotrophic functions and so also their tumourigenic deviations are related to certain cell types as shown especially by the outstanding experimental work of Furth (1954) and his colleagues on thyrotrophin- or corticotrophin-secreting pituitary tumours. But production of suprarenal tumours by castration as known from classic work of Woolley and others, and the possibility of controlling the tumourigenic deviation of the gonadotrophic function of the hypophysis by cortisone—should our tentative suggestion be right that cortisone has acted not on the tumour itself—show that the organotrophic hypophysial potency is an *integrated whole* and that different organotrophic functions of the hypophysis are *intertwined* in the narrow space of the anterior lobe (see also Section 11 in Lipschutz, 1956).

SUMMARY

Cortisone acetate was administered to castrated mice of two different strains (C57bl and Balb A) bearing intrasplenic ovarian grafts, to study the question whether metastatic spread of a tumour growing under the given experimental conditions could be enhanced by this steroid. Cortisone was allowed to act 4 to 6 months through continuous absorption from subcutaneously implanted pellets.

Metastatic spread was not enhanced in the cortisone animals.

* Neither is cortisone an antitumourigenic steroid *per se* ! Survival and proliferation of human tumours transplanted into rats is rendered possible by the administration of cortisone (Toolan, 1953).

The quantities of cortisone acetate administered greatly slowed down the growth of the intrasplenic ovarian tumours in both strains used.

The microscopical structure of the tumours also was fundamentally influenced by cortisone : the incidence of granulosa-cell tumours greatly diminished and the incidence of luteomata plus luteomatous nodules increased.

The small luteomatous ovarian growths, tumoural or non-tumoural, in the two strains of mice treated with cortisone were similar to the slowly growing intrasplenic ovarian growths in the guinea-pig in the first two years after grafting.

The tentative suggestion is made that the antitumourigenic action of cortisone was by interference with the production or delivery of gonadotrophic hypophysial hormones on which ovarian tumourigenesis in intrasplenic grafts depends.

If this interpretation were true the results obtained would suggest that differences as to the evolution of intrasplenic ovarian tumours between guinea-pigs and mice, or between different strains of mice, should be explained by a genetically determined difference in the pattern of the steroid homeostasis.

The results obtained would likewise offer a new example of how a tumourigenic deviation of the *gonadotrophic* function of the hypophysis can be influenced by a corticoid whose production in the body is dependent on the *corticotrophic* function of the hypophysis.

Cordial thanks are due especially to Miss Socorro Salinas for generous help at operations and necropsies, and to Mr. Alejandro Castillo, photographer.

### REFERENCES

AGOSIN, M., CHRISTEN, R., BADINEZ, O., GASIC, G., NEGHME, A., PIZARRO, O. AND JARPA, A.—(1952) *Proc. Soc. exp. Biol.*, N.Y., **80**, 128.
ANTOPOL, W., GLAUBACH, S. AND GRAFF, S.—(1954) *Ibid.*, **86**, 364.
BASERGA, R. AND SHUBIK, P.—(1954) *Cancer Res.*, **14**, 12.
BISKIND, M. S. AND BISKIND, G. R.—(1944) *Proc. Soc. exp. Biol., N.Y.*, **55**, 176.
FUENZALIDA, F.—(1950) *J. clin. Endocrin.*, **10**, 1511.
*Idem* AND LIPSCHUTZ, A.—(1953) *Ibid.*, **13**, 1201.
FURTH, J.—(1954) *Amer. J. Path.*, **30**, 42.
*Idem* AND SOBEL, H.—(1947) *J. nat. Cancer Inst.*, **8**, 7.
GARDNER, W. U.—(1955) *Cancer Res.*, **15**, 109.
IGLESIAS, R., LIPSCHUTZ, A. AND MARDONES, E.—(1950) *J. Endocrin.*, **6**, 365.
*Idem*, MARDONES, E. AND LIPSCHUTZ, A.—(1953*a*) *Brit. J. Cancer*, **7**, 214.—(1953*b*) *Ibid.*, **7**, 221.
KALISS, N., BORGES, P. R. F. AND DAY, E. D.—(1954) *Cancer Res.*, **14**, 210.
KLEIN, M.—(1952) *J. nat. Cancer Inst.*, **12**, 877.—(1953) *Ibid.*, **14**, 77.
LI, M. H.—(1948) *Amer. J. Obstet, Gynec.*, **55**, 316.
*Idem* AND GARDNER, W. U.—(1947) *Cancer Res.*, **7**, 549.—(1949) *Ibid.*, **9**, 35.
LIPSCHUTZ, A.—(1956) 'Steroid Homeostasis, Hypophysis and Tumourigenesis.' Cambridge (Heffer & Sons).
*Idem*, PONCE DE LEÓN, H., WOYWOOD, E. AND GAY, O.—(1946) *Rev. canad. Biol.*, **5**, 181.
MARDONES, E., IGLESIAS, R. AND LIPSCHUTZ, A.—(1955) *Brit. J. Cancer*, **9**, 409.—(1956) *Endocrinology*, **58**, 212.
MARTÍNEZ, C. AND BITTNER, J. J.—(1955) *Proc. Soc. exp. Biol., N.Y.*, **89**, 569.
MOLOMUT, N., SPAIN, D. M., GAULT, S. D. AND KREISLER, L.—(1952) *Proc. nat. Acad. Sci.*, **38**, 991 (quoted from Martínez and Bittner).
POMEROY, T. C.—(1954) *Cancer Res.*, **14**, 201.
SHIMKIN, M. B.—(1955) *Advanc. Cancer Res.*, **3**, 223.
TOOLAN, H. W.—(1953) *Cancer Res.*, **13**, 389.